# Benefits of Initial Limited Crystalloid Resuscitation in Severely Injured Trauma Patients at Emergency Department

**DOI:** 10.14740/jocmr2355w

**Published:** 2015-10-23

**Authors:** Hao Wang, Richard D. Robinson, Jessica Laureano Phillips, Alexander J. Kirk, Therese M. Duane, Johnbosco Umejiego, Melanie Stanzer, Mackenzie B. Campbell-Furtick, Nestor R. Zenarosa

**Affiliations:** a Department of Emergency Medicine, Integrative Emergency Services Physician Group, John Peter Smith Health Network, 1500 S. Main St., Fort Worth, TX 76104, USA; b Department of Surgery, John Peter Smith Health Network, 1500 S. Main St., Fort Worth, TX 76104, USA

**Keywords:** Crystalloid, Packed red blood cell, Resuscitation, Emergency department, Trauma

## Abstract

**Background:**

Whether initial limited crystalloid resuscitation (LCR) benefits to all severely injured trauma patients receiving blood transfusions at emergency department (ED) is uncertain. We aimed to determine the role of LCR and its associations with packed red blood cell (PRBC) transfusion during initial resuscitation.

**Methods:**

Trauma patients receiving blood transfusions were reviewed from 2004 to 2013. Patients with LCR (L group, defined as < 2,000 mL) and excessive crystalloid resuscitation (E group, defined as ≥ 2,000 mL) were compared separately in terms of basic demographic, clinical variables, and hospital outcomes. Logistic regression, R-square (R^2^), and Spearman rho correlation were used for analysis.

**Results:**

A total of 633 patients were included. The mortality was 51% in L group and 45% in E group (P = 0.11). No statistically significant difference was found in terms of basic demographics, vital signs upon arrival at ED, or injury severity between the groups. The volume of blood transfused strongly correlated with the volume of crystalloid infused in E group (R^2^ = 0.955). Crystalloid to PRBC (C/PRBC) ratio was 0.8 in L group and 1.3 in E group (P < 0.01). The correlations between C/PRBC and ED versus ICU versus hospital length of stay (LOS) via Spearman rho were 0.25, 0.22, and 0.22, respectively.

**Conclusions:**

Similar outcomes were observed in trauma patients receiving blood transfusions regardless of the crystalloid infusion volume. More crystalloid infusions were associated with more blood transfusions. The C/PRBC did not demonstrate predictive value regarding mortality but might predict LOS in severely injured trauma patients.

## Introduction

While severely injured trauma patients account for a relatively small percent of overall ED presentations, their injuries result in higher in-hospital mortality as well as longer in-hospital length of stay (LOS) [1-3]. Hemorrhagic shock is the leading cause of early mortality in these patients [4, 5]. According to the current advanced trauma life support (ATLS) guidelines, fluid resuscitation with both crystalloid infusion and blood transfusion remains the mainstay of initial treatment in severely injured trauma patients (high risk bleeding potential per treating clinician interpretation). Further surgical intervention is considered if patients fail to adequately respond to fluid resuscitation. Recent trauma studies indicate that performing damage control resuscitation (DCR) in severely injured trauma patients is associated with a higher survival rate and shorter in-hospital LOS [6-8]. Permissive hypotension, early hemostatic resuscitation with blood products, and restriction of crystalloid infusion are now recommended during the initial resuscitation of these patients. Concomitantly, evidence linking “excessive” crystalloid resuscitation to worsening clinical outcomes including higher in-hospital mortality, prolonged intensive care unit (ICU) stay, increased complication rates (e.g., acute abdominal compartment syndrome, acute respiratory distress syndrome, etc.), and higher rates of mechanical ventilation has been reported in several studies [9-11]. Of note the findings of these studies were mainly reported in trauma patients requiring massive transfusions (MTs) with different amounts of “excessive” crystalloid infusions; therefore, uncertainty remains as to whether the same initial treatment strategy can be extended to all severely injured trauma patients requiring both crystalloid infusion and blood transfusion in the emergency department (ED) [12-14].

In addition, previous studies have identified the crystalloid infusion to packed red blood cell transfusion ratio (C/PRBC) as a marker that may be associated with, and therefore potentially predictive of, the severity of injury, hospital complications, and general prognosis associated with severely injured trauma patients [15-17]. This C/PRBC ratio was calculated based on the amount of total crystalloid infused initially and the number of units of PRBC received in trauma patients during their initial ED resuscitations. A higher ratio of crystalloid to PRBC transfusion was previously found to be associated with high risk of hospital complications and prolonged LOS [16]. However, the initial ED resuscitation C/PRBC ratio was also monitored in patients requiring MT and the findings in different studies were controversial [18]. Currently uncertainty remains as to whether an optimal level of crystalloid restriction during initial resuscitation should be pursued, whether C/PRBC ratio can be used to determine the appropriate level of crystalloid resuscitation, and whether these recommendations can be extended to all severely injured trauma patients.

Acute trauma patients who received both crystalloid infusion and blood transfusion during initial ED resuscitation are assumed to have severe trauma-related injuries, high incidence of hemorrhage and increased hospital mortality [19, 20]. ED management of these patients focuses on early stabilization which largely includes crystalloid and blood products administration. In this study, we defined trauma patients who received both crystalloid infusion and blood transfusion in the ED as severely injured. We sought to determine: 1) whether excessive crystalloid infusion is necessary before/during blood transfusion; and 2) whether the C/PRBC ratio can be used to guide initial fluid resuscitation and predict outcomes.

## Methods

### Selection of participants

Retrospective review of local trauma registry data for the period January 2004 through December 2013 was performed. Analysis included data associated with adult trauma patients (≥ 18 years) presenting to the study center ED that received both crystalloid infusion and blood transfusion resuscitation. Patients whose age was unknown and those < 18 years of age, those initially presenting to the ED without need of blood transfusion, and those with missing or unknown data regarding crystalloid volume received during their ED stay were excluded from this study. Since this study focused on the association of crystalloid infusion, blood transfusion, and their outcome measurements, patients who expired prior to hospital admission (i.e. dead on arrival (DOA)), were also excluded.

### Study design and protocol

Patients were included in the study and classified as having received crystalloid infusion if they received either normal saline (NS) or lactated ringers (LRs) while in the ED. As our trauma registry only records emergent transfusions, which per our protocols include only uncross-matched PRBC transfusions, all uncross-matched transfusions were included in the cohort that received blood transfusions. Calculations comparing volumes of intravenous crystalloid and PRBCs administered included both pre-hospital amounts and those given in the ED prior to admission as ED resuscitation totals.

Patient outcomes such as all-cause in-hospital mortality (hereafter referred to as mortality) and LOS were calculated. Mortality was divided into early (defined as confirmed death within 24 h of hospital arrival) and late (defined as confirmed death in greater than 24 h of hospital arrival). ED, ICU, and in-hospital LOS were measured and compared separately. In order to determine whether the amount of crystalloid resuscitation impacted outcomes, patients who received ED blood transfusions were categorized into different groups. Different amounts of crystalloid that patients received at ED were used as different thresholds in different groups for mortality comparisons (including 500 mL, 1,000 mL, 1,500 mL, 2,000 mL, 4,000 mL, and 6,000 mL). In addition, as a 2,000 mL of crystalloid infusion was recommended during initial resuscitation by classic ATLS, an extensive analysis was performed. Patients who received less than 2,000 mL of crystalloid before leaving the ED (prehospital and in-ED total) were further placed in the “L group” (less crystalloid resuscitation). Those patients who received 2,000 mL or more of crystalloid were included in the “E group” (excessive crystalloid resuscitation). Basic patient demographics (age, sex, race/ethnicity, and mode of arrival), ED clinical variables (initial vital signs, injury severity using both a revised trauma severity score and injury severity score, Glasgow coma scale (GCS), crystalloid infusion and blood transfusion volumes) and ED disposition (e.g. admitted to ICU or transferred directly to operating rooms (ORs)) were analyzed and compared between these two groups. In order to investigate the trend of initial trauma resuscitation efficiency in line with the patient mortality and injury severity in the past 10 years, the amounts of crystalloid infusion and blood transfusion volumes were also analyzed and compared in each year separately.

To determine the associations of crystalloid infusion and blood transfusion, the correlation between the volume of crystalloid infusion and the number of units of PRBC transfusion was analyzed. Additionally, C/PRBC ratio was calculated to determine the appropriateness of balanced crystalloid and blood resuscitations. C/PRBC was defined as the ratio of crystalloid infused in liters to the units of PRBCs transfused. The correlations between C/PRBC and ED, ICU, and in-hospital LOS were analyzed. The local institutional review board approved this study.

### Data analysis

Student’s *t*-test was used to compare continuous variables between two groups, while analysis of variance with Bonferroni correction was used to analyze differences among groups. Pearson Chi-square (χ^2^) analysis was used to compare categorical variables. To control for confounders, independent clinical variables were entered into a multivariate logistic regression model. R-square (R^2^) analysis estimated the proportion of variance in the dependent variable that is accounted for by the independent variable and was used to determine the strength of the relationship between the volume of crystalloid infusion and blood transfusion at ED. R^2^ > 0.8 was considered strong correlation. Categorizing patients into two subgroups (L versus E) may have distorted the distribution. To account for this, Spearman’s rho (ρ) correlation was used to determine the potential correlation between variables for which non-normal distribution may have occurred. Strength of relationships was determined as follows: 1) strong correlations were ρ > 0.5, 2) moderate correlations were between 0.2 and 0.5, and 3) values < 0.2 were considered weak correlations. Receiver operating characteristic (ROC) curve and the area under the ROC (AUC) were measured to determine the accuracy of C/PRBC ratio to predict in-hospital mortality between L and E groups. All descriptive and statistical analyses were conducted using Stata 12.0 (College Station, TX). A P value less than 0.05 was considered statistically significant.

## Results

**Figure 1:**
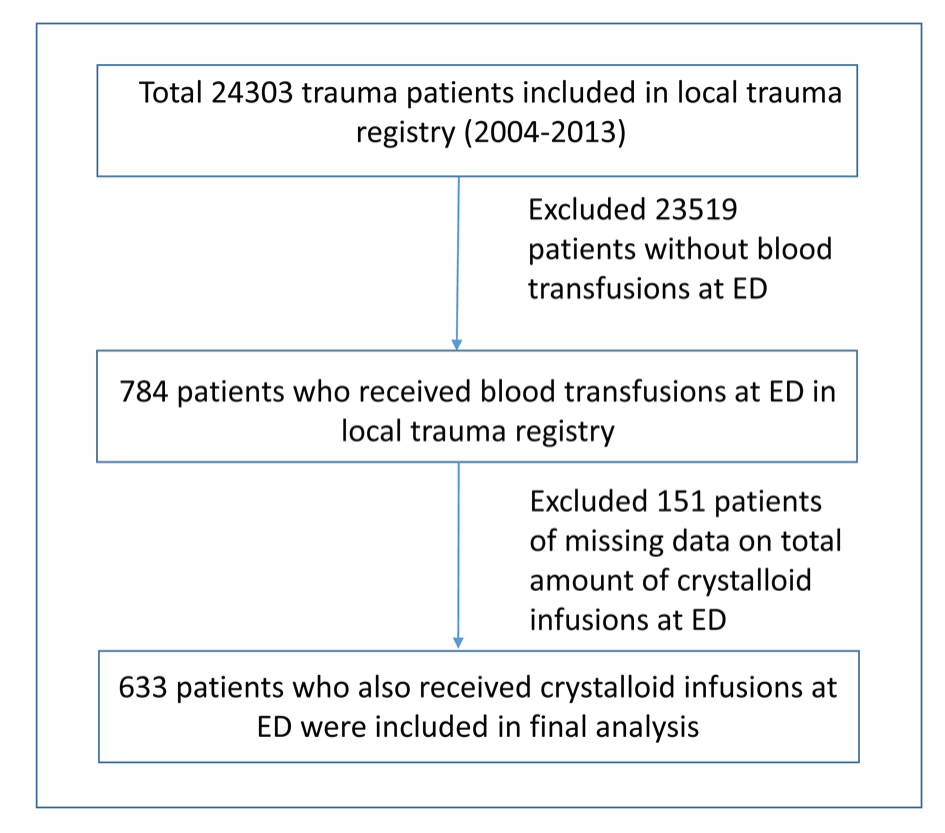
The flow diagram of patients placed in final analysis.

Initially, 24,303 trauma patients were reviewed in our local trauma registry over the period January 1, 2004 through December 31, 2013. Among these patients, 784 received blood transfusions in the ED accounting for 3.2% of total trauma patients during the period of interest. Since this study was intended to compare clinical outcomes in patients who received both crystalloid infusions and blood transfusions in the ED, 151 patients with missing information on the total volume of intravenous crystalloid fluid infusions at ED were excluded from the study resulting in a missing data rate of 19.2% (151/784). A total of 633 patients were entered into the final analysis (Fig. 1).

**Figure 2:**
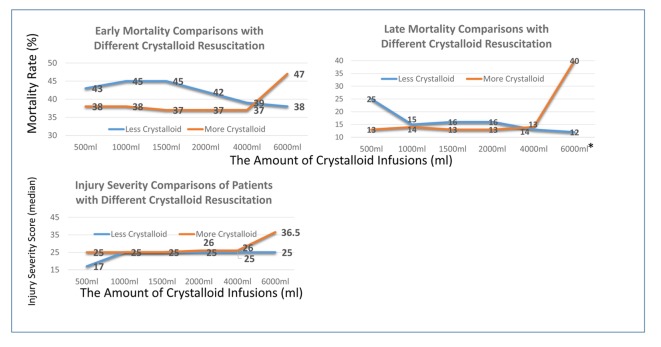
The comparisons of mortality between patients receiving different amounts of crystalloid resuscitations. *Statistically significant difference between two groups (P < 0.01).

**Figure 3:**
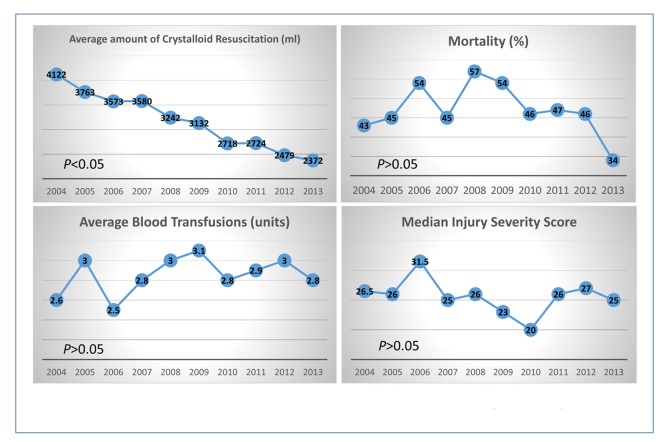
The comparisons of the amount of crystalloid resuscitation, blood transfusion volume, mortalities, and injury severity scores in trauma patients each year from 2004 until 2013.

No significant difference was found in terms of early and late mortality when patients received different amounts of crystalloids except those who received more than 6,000 mL of crystalloid infusions at ED (Fig. 2). Moreover, similar injury severities were found between groups except in groups of patients receiving less than 500 mL or more than 6,000 mL of crystalloid. This also resulted in a higher late mortality in group of patients receiving more than 6,000 mL of crystalloid than those without (40% versus 12%, P < 0.01). The amounts of crystalloid infusions and blood transfusions were also investigated separately in each year (2004 - 2013); our findings showed the trend of decreased crystalloid usage while the blood transfusion volumes have not been changed (Fig. 3). Meanwhile, though the mortality tended to improve in recent years in patients with similar injury severity, no statistical significant difference was reached (Fig. 3).

Furthermore, 210 patients who received < 2,000 mL crystalloid fluid infusion before leaving the ED were compared with the remaining 423 patients who received ≥ 2,000 mL of crystalloid. All patients received blood transfusions at ED before the final dispositions. General patient demographics and clinical variables showed no statistically significant difference between the two groups, with the exception of age and systolic blood pressure while multivariate logistic regression analysis identified both as confounders without any clinically or statistically significant differences between groups (Supplementary 1, www.jocmr.org).

ED variables including injury severity score, vital signs, GCS, hemoglobin, and ED disposition also showed no difference between groups (Table 1). The average crystalloid infusion volume in L group was 1,352 mL, whereas E group averaged 3,870 mL infused crystalloid volume (P < 0.01). Blood transfusions showed a statistically significant difference in univariate analysis but reached no difference in multivariate analysis (Supplementary 1, www.jocmr.org). ICU and OR disposition yielded similar results in the two groups, though a higher rate of OR transfer that occurred in L group, P > 0.05 (Table 1). Among all trauma patients transferred to the OR, blood transfusion volume was independent risk predictive of crystalloid resuscitation volume in both univariate and multivariate logistic regression analysis (Table 1 and Supplementary 1, www.jocmr.org).

**Table 1:** A Comparison of Crystalloid Fluid Resuscitation Among Pelvic Patients Receiving Blood Transfusions in Emergency Department

	Patients who received < 2,000 mL crystalloid (N = 210)	Patients who received ≥ 2,000 mL crystalloid (N = 423)	P value
General demographics			
Age (years)	38.09 ± 16.46	41.03 ± 18.19	0.05
Gender (male, %)	80.95%	77.54%	0.32
Race (white, %)	52.38%	59.57%	0.26
Mode of arrival (ambulance/helicopter, %)	92.86%	95.98%	0.09
ED clinical variables			
RTS on arrival	4.66 ± 2.81	4.96 ± 2.62	0.22
ISS on arrival	25.5 ± 18.7	28.5 ± 17.6	0.05
ED SBP (mm Hg, mean ± SD, 95%CI)	81 ± 48 (74 - 88)	87 ± 42 (83 - 91)	0.13
ED HR (bpm, mean ± SD, 95% CI)	93 ± 48 (87 - 100)	96 ± 43 (92 - 100)	0.52
ED RR (times, mean ± SD, 95% CI)	14 ± 9 (13 - 16)	15 ± 9 (14 - 16)	0.66
GCS (mean ± SD)	8.0 ± 5.4 (7.3 - 8.8)	8.3 ± 5.4 (7.8 - 8.8)	0.58
Hgb (mg/dL, mean ± SD)	11.3 ± 2.5 (10.9 - 11.7)	11.1 ± 2.4 (10.8 - 11.3)	0.29
ED total crystal fluid received (mL, mean ± SD)	1,352 ± 621 (1,267 - 1,437)	3,870 ± 1,419 (3,734 - 4,006)	< 0.01
ED blood transfusion (units, mean ± SD)	2.53 ± 1.51 (2.32-2.73)	3.01 ± 1.97 (2.82 - 3.20)	< 0.01
ED total LOS (h, mean ± SD)	0.8 ± 1.2 (0.7-1.0)	1.3 ± 1.8 (1.1 - 1.5)	< 0.01
C/PRBC ratio (ratio, mean ± SD)	0.71 ± 0.54	1.70 ± 1.02	< 0.01
In-hospital variables			
Mortality (n, %)	108/210 (51.43%)	189/423 (44.68%)	0.11
Early mortality (n, %)	89/108 (82.41%)	155/189 (82.01%)	0.93
ICU LOS (days, mean ± SD, 95% CI))	7.0 ± 11.4 (5.5 - 8.6)	9.2 ± 13.0 (8.0 - 10.5)	0.04
Hospital LOS (days, mean ± SD, 95% CI)	9.7 ± 15.4 (7.6 - 11.8)	12.3 ± 16.4 (10.7 - 13.9)	0.06

RTS: revised trauma score; ISS: injury severity score; SBP: systolic blood pressure; GCS: Glasgow coma scale; LOS: length of stay; C/PRBC: crystalloid to packed red blood cell ratio; CI: confidence interval.

In-hospital early and late mortalities showed no significant difference between L and E groups, regardless of the volume of crystalloid infusion received (Table 2). C/PRBC ratio was used as a resuscitation marker. L group had an average ratio of 0.8 versus 1.3 in E group (P = 0.0012) (Table 2). We found similar results when patients were further divided into different ED dispositions (e.g. ICU versus OR, Table 2). This analysis yielded no significant difference for in-hospital mortality in patients of different C/PRBC ratios (Table 2). C/PRBC ratio AUC predicting mortality in L group was 0.3849 (95% CI: 0.3083 - 0.4614) versus 0.3509 (95% CI 0.2981 - 0.4035) in E group and was therefore non-discriminate. Though not statistically significant, patients with a low C/PRBC ratio tended to have shorter ICU and in-hospital LOS (Table 2).

**Table 2:** A Comparison of Outcome Measurements in Trauma Patients Who Received Different Crystalloid Resuscitation at Emergency Department

	Patients who received < 2,000 mL crystalloid	Patients who received ≥ 2,000 mL crystalloid	P value
Total trauma patients at ED	210	423	
C/PRBC ratio (ratio, mean ± SD)	0.71 ± 0.54	1.70 ± 1.02	< 0.01
Mortality (n, %)	108/210 (51%)	189/423 (45%)	0.11
Early mortality (n, %)	89/108 (82%)	155/189 (82%)	0.93
Late mortality (n, %)	19/108 (16%)	34/189 (18%)	0.93
ED total LOS (h, mean ± SD)	0.8 ± 1.2 (0.7 - 1.0)	1.3 ± 1.8 (1.1-1.5)	< 0.01
ICU LOS (days, mean ± SD, 95% CI))	7.0 ± 11.4 (5.5 - 8.6)	9.2 ± 13.0 (8.0-10.5)	0.04
Hospital LOS (days, mean ± SD, 95% CI)	9.7 ± 15.4 (7.6 - 11.8)	12.3 ± 16.4 (10.7-13.9)	0.06
ED admitted to ICU	63/210 (30%)	177/423 (42%)	
C/PRBC ratio (ratio, mean ± SD)	0.76 ± 0.60	1.80 ± 1.06	< 0.01
Mortality (n, %)	26/63 (41%)	66/177 (37%)	0.58
Early mortality (n, %)	16/26 (62%)	46/66 (70%)	0.45
Late mortality (n, %)	10/26 (38%)	20/66 (30%)	0.45
ED total LOS (h, mean ± SD)	1.6 ± 1.8 (1.1 - 2.0)	1.5 ± 1.3 (1.3 - 1.7)	0.58
ICU LOS (days, mean ± SD, 95% CI))	10.04 ± 12.71	12.06 ± 15.66	0.36
Hospital LOS (days, mean ± SD, 95% CI)	12.55 ± 17.60	16.15 ± 20.30	0.21
ED transferred to OR	111/210 (53%)	187/423 (44%)	
C/PRBC ratio (ratio, mean ± SD)	0.72 ± 0.52	1.64 ± 0.99	< 0.01
Mortality (n, %)	51/111 (46%)	79/187 (42%)	0.53
Early mortality (n, %)	42/51 (82%)	65/79 (82%)	0.99
Late mortality (n, %)	9/51 (18%)	14/79 (18%)	0.99
ED total LOS (h, mean ± SD)	0.4 ± 0.4 (0.3 - 0.5)	0.8 ± 0.8 (0.7 - 1.0)	< 0.01
ICU LOS (day, mean ± SD, 95% CI)	7.63 ± 11.74	9.12 ± 11.04	0.27
Hospital LOS (days, mean ± SD, 95% CI)	11.00 ± 15.81	11.82 ± 13.26	0.63

**Figure 4:**
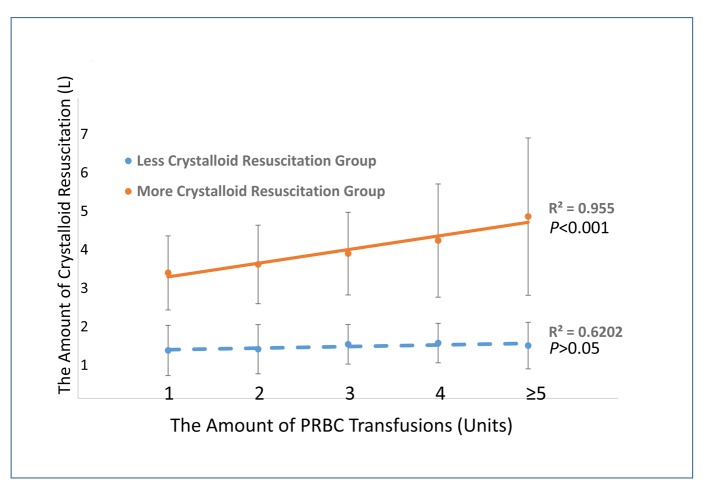
The association between crystalloid resuscitation and PRBC transfusions in ED trauma patients of different groups.

**Figure 5:**
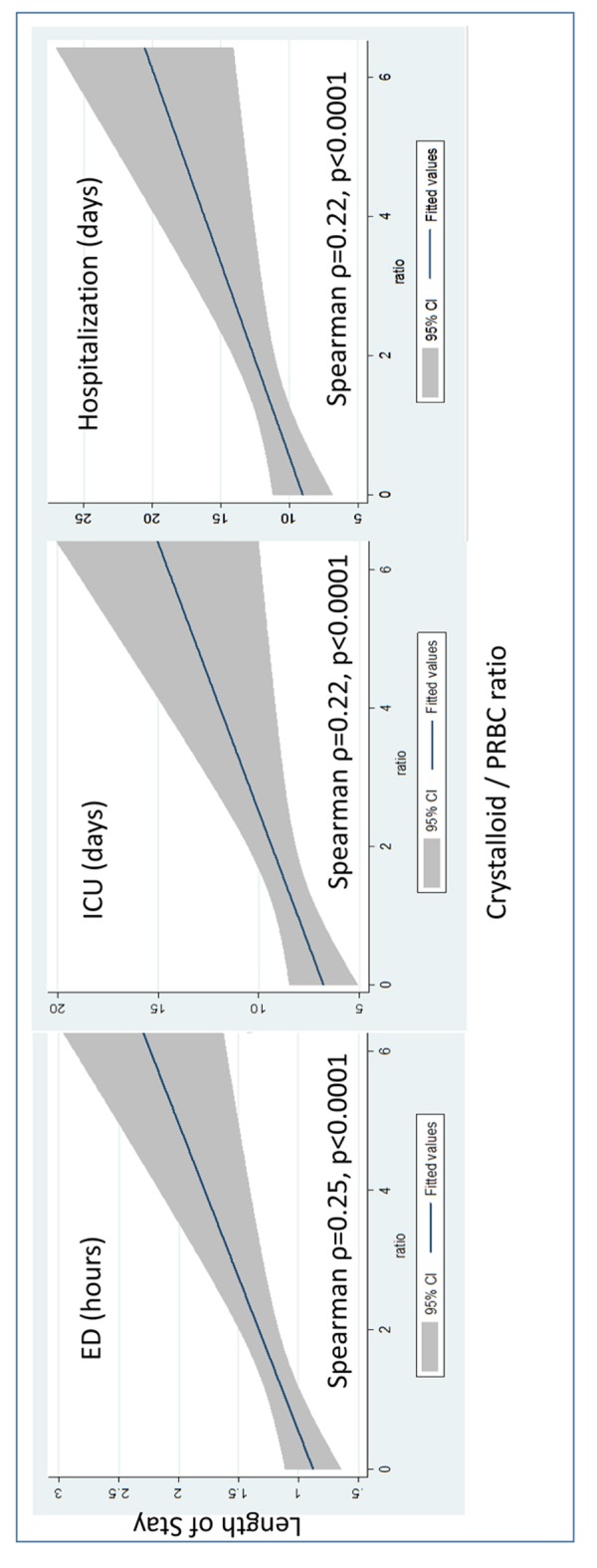
The association of C/PRBC ratio and the length of ED, ICU, and hospitalization using confidence band around regression prediction.

Our results showed no significant difference of crystalloid resuscitation in L group, despite blood transfusion volume received in the ED (P > 0.05). However, the volume of crystalloid given in E group was strongly correlated with the blood transfusion volume (R^2^ = 0.955, P < 0.05) (Fig. 4). Moderate correlations were found between C/PRBC ratios and ED, ICU, and in-hospital LOS among all trauma patients in this study (Fig. 5).

## Discussion

Given the relatively high in-hospital mortality, especially during the first 24 h of care, it is critical to properly resuscitate severely injured trauma patients while in the ED. In recent years, damage control resuscitation has been well studied and suggests better outcomes with lower volume crystalloid infusion and early blood product transfusion for severely injured trauma patients. Differences remain between the findings of evidence-based studies and current ATLS recommendations for standard resuscitations, especially in regard to initial fluid infusion [21, 22]. Our study showed that acute trauma patients requiring blood transfusions in the ED who also received low volume crystalloid infusion had no inferior outcomes when compared with those who received excessive crystalloid infusion during ED resuscitation. In addition, these patients also tended to have better balanced resuscitation resulting in shorter ICU and in-hospital LOS. The results of this study may provide evidence to the literature pool on limited crystalloid resuscitation in severely injured trauma patients. It would be worthwhile to revisit the current trauma resuscitation algorithm in ATLS and extend DCR treatment strategy to all severely injured trauma patients.

As mentioned above, the benefits of limited crystalloid resuscitation in trauma patients receiving MT include decreased mortality, in-hospital complications, fewer surgical interventions, and shortened hospital LOS [8, 9, 12]. In recent years, these benefits have been found in trauma patients in the pre-hospital setting regardless of MT. Studies on pre-hospital crystalloid resuscitation also show decreased early in-hospital mortality in patients receiving limited crystalloid [23, 24]. Duggan reported similar benefits extended to non-trauma patients experiencing active gastrointestinal bleeding [25]. Considering the similar mechanism of increasing coagulopathy occurring in the early stage in patients with severe trauma, extending a limited crystalloid resuscitation strategy to these patients requiring blood transfusions seems reasonable. This was also seen as a trend in recent clinical practice in the study ED as well (Fig. 3). On the other hand, given the evidence that physician gestalt has poor sensitivity and specificity in predicting the need for MT [26], it is unrealistic to determine initially, or to even predict during the early stages of a resuscitation, whether trauma patients will ultimately require MT upon arrival to the ED. In addition, it is easier to follow the pattern of giving more crystalloid when higher volume blood transfusions are administered to trauma patients during ED resuscitation (Fig. 4, E group), whereas this pattern does not seem significant when patients received limited crystalloid. Thus special attention should be paid to avoiding excessive crystalloid infusions in severely injured trauma patients requiring significant blood transfusion volumes.

Simply reviewing the volume of crystalloid without considering the volume of blood transfusion at the same time might cause biased results. Therefore, it is important to determine and dynamically monitor whether severely injured trauma patients receive balanced fluid resuscitations. Originally, the C/PRBC ratio was applied to severely injured trauma patients requiring MT. This resuscitation marker showed patients with high C/PRBC ratios tended to have higher in-hospital complications and longer hospital LOS whereas mortality was not affected [16]. However, those results were not significant when applied to all trauma patients receiving PRBC [18, 27]. Our study extends the use of C/PRBC to all severely injured trauma patients and validates previous findings for which C/PRBC neither affects nor predicts in-hospital mortality regardless as to which cut off points were used. However, it did show the trend of prolonged ICU and in-hospital LOS, with weak to moderate correlation in this study, yet no statistically significant difference was reached. This may indirectly be attributed to the increased in-hospital complications which occur with excessive crystalloid resuscitation [18, 28]. Due to the limited data in this study, we were not able to address this association accurately. Future prospective research should focus on the potential risk factors predictive of ED blood transfusions and the role of the C/PRBC ratio in predicting certain in-hospital complications among all trauma patients.

### Limitations

Retrospective study designs cannot demonstrate causality due to limited information accuracy, missing data, and potential selection bias. A small sample size and large group distribution difference can generate bias and make statistical analysis less powerful. We are not able to include all different clinical variables for data analysis such as in-hospital complications, direct cause of death, or prehospital transportation time. Based on historical data in our local trauma center database, prehospital transportation time is relatively low (i.e. a matter of minutes), making this confounder less affective. Intentionally dividing patients into two groups (L or “less” and E or “excessive” crystalloid infusion groups) can potentially generate some variables not normally distributed such as the volume of crystalloid resuscitation, the volume of blood transfusion, and C/PRBC ratios. In addition, this also indicates that the missing data were most likely not distributed randomly making data attribution impossible.

### Conclusions

Our study shows similar outcomes in severely injured trauma patients who received limited crystalloid resuscitation when compared with patients receiving excessive crystalloid resuscitation. In addition, more blood transfusions were associated with more crystalloid infusions. While C/PRBC ratio was unable to predict in-hospital mortality, it may be useful in predicting in-hospital LOS among all severely injured trauma patients.
